# Intersections between rural women’s resilience and quality of life: a mixed-methods study ^*^

**DOI:** 10.1590/1518-8345.5671.3521

**Published:** 2022-04-29

**Authors:** Bruno Neves da Silva, José Luís Guedes dos Santos, Deise Lisboa Riquinho, Francisco Arnoldo Nunes de Miranda, Nilba Lima de Souza, Erika Simone Galvão Pinto

**Affiliations:** 1 Universidade Federal do Rio Grande do Norte, Natal, RN, Brasil.; 2 Universidade Federal de Santa Catarina, Florianópolis, SC, Brasil.; 3 Universidade Federal do Rio Grande do Sul, Escola de Enfermagem, Porto Alegre, RS, Brasil.

**Keywords:** Women, Rural Areas, Psychological Resilience, Quality of Life, Rural Health, Health Promotion, Mulheres, Zona Rural, Resiliência Psicológica, Qualidade de Vida, Saúde da População Rural, Promoção da Saúde, Mujeres, Medio Rural, Resiliencia Psicológica, Calidad de Vida, Salud Rural, Promoción de la Salud

## Abstract

**Objective:**

to analyze the intersections between rural women’s quality of life and resilience.

**Method:**

convergent mixed methods design in which a cross-sectional quantitative study is triangulated with a qualitative study guided by Oral History. Data were collected concomitantly, using a socio-demographic form, Resilience Scale, Medical Outcomes Study 36-Item Short-Form Health Survey, and open-ended interviews. The analysis was based on descriptive and inferential statistics and inductive thematic analysis, which was integrated later.

**Results:**

an association was found between the social aspects domain of quality of life and a moderate level of resilience related to the characteristics of life in rural areas. The integration of results enabled verifying that these two constructs (which mutually influence each other) are mediated by protective factors, resilience developed by the rural women, such as spirituality and the formation of social support, enchantment, and a feeling of belonging to their context.

**Conclusion:**

by developing protective factors, rural women develop a resilient behavior that favors their quality of life. Identifying these factors enables the development of psychosocial interventions to promote rural women’s health.

## Resumo

**Objetivo:**

analisar as interseções entre qualidade de vida e resiliência de mulheres rurais.

**Método:**

estudo de métodos mistos convergente, no qual foram triangulados um estudo quantitativo de corte transversal e um estudo qualitativo guiado pela da história oral de vida. Os dados foram concomitantemente coletados utilizando-se de um formulário sociodemográfico, da Escala de Resiliência, do *Medical Outcomes Study 36-Item Short-Form Health Survey* e mediante entrevistas abertas. A análise deu-se por estatística descritiva e inferencial e análise temática indutiva, com posterior integração.

**Resultados:**

constatou-se associação entre o domínio aspectos sociais de qualidade de vida e o grau moderado de resiliência, relacionado às características do cotidiano rural. A integração dos resultados possibilitou reconhecer que a interseção entre os dois construtos, que se influenciam mutuamente, ocorre pela mediação de fatores de proteção para resiliência elaborados pelas mulheres rurais, como a espiritualidade, a formação de redes de apoio social e os sentimentos de encantamento e pertencimento ao seu contexto.

**Conclusão:**

a partir da elaboração de fatores de proteção, as mulheres rurais desencadeiam atitudes resilientes que contribuem para sua qualidade de vida. A identificação desses fatores permite o desenvolvimento de intervenções psicossociais que podem contribuir para a promoção da saúde da mulher rural.

## Resumen

**Objetivo:**

analizar las intersecciones entre calidad de vida y resiliencia en mujeres rurales.

**Método:**

estudio de métodos mixtos convergentes, en el que fueron triangulados un estudio cuantitativo de corte transversal y un estudio cualitativo guiado por la historia oral de vida. Los datos fueron concomitantemente recolectados utilizando formulario: sociodemográfico de la Escala de Resiliencia y del *Medical Outcomes Study 36-Item Short-Form Health Survey;* y, mediante entrevistas abiertas. El análisis se realizó con la estadística descriptiva e inferencial, y con el análisis temático inductivo, con posterior integración.

**Resultados:**

se constató asociación entre el dominio aspectos sociales de calidad de vida y el grado moderado de resiliencia, relacionado con las características de lo cotidiano rural. La integración de los resultados posibilitó reconocer que la intersección entre los dos constructos, que se influencian mutuamente, ocurre por la mediación de factores de protección para la resiliencia, elaborados por las mujeres rurales, como la espiritualidad, la formación de redes de apoyo social, y los sentimientos de encantamiento y pertenencia a su contexto.

**Conclusión:**

a partir de la elaboración de factores de protección, las mujeres rurales desencadenan actitudes resilientes que contribuyen para su calidad de vida. La identificación de esos factores permite el desarrollo de intervenciones psicosociales que pueden contribuir para la promoción de la salud de la mujer rural.

**Highlights:**

(1) The quality of life of rural women is influenced by their level of resilience.

(2) Rural women develop protective factors that result in resilience.

(3) Rural women’s resilience influences their quality of life.

(4) The approach of a mixed method design validates the results

## Introduction

A rural woman is a female living in the rural area, whose everyday life is characteristic of this context, with a family structure and lifestyle typical to the life spent in rural areas, spending her time in reproductive and housework and farming, i.e., agriculture^1^.

Although advancements were achieved in public policy, such as rural retirement and access to credit, the singularity of rural women is marked by socioeconomic hardships and behavioral and health problems arising from their context, which directly interfere with their quality of life (QoL)^2^. Historically, the sociocultural processes in Brazilian rural areas assign different roles to genders, impeding women from playing a productive role, i.e., they are considered helpers of their fathers and husbands in agriculture income-generating activities but are essential for reproductive activities and housework^3^. 

The lives of women in rural areas are marked by the environment where they live, while living and work conditions directly influence their health, exposing them to the risk of diseases. Gender-related aspects also result in more negative QoL among rural women^4^. According to the World Health Organization, QoL is a subjective and multidimensional construct that includes biopsychosocial aspects, beliefs, independence level, environmental characteristics, and social relationships^5^. 

Even though women’s work in rural areas is often overlooked, they do not lose their perseverance and courage and keep enduring, seeking improved living conditions supported by resilience processes. Resilience is a concept that represents an individual’s flexibility and ability to bring resources to adapt to adverse situations, aiming to maintain a biopsychological balance^6^. 

This study enables unveiling aspects related to the QoL of rural women, considering contextual inequalities that affect these women’s history of life and their health-disease-care process. It also contributes to developing interventions and public policies to promote improved QoL and, consequently, rural women’s health, which should be considered a more comprehensive phenomenon than diseases. From this perspective, improved QoL represents one of the most difficult challenges imposed on public policies directed to populations in rural areas^7^.

QoL and resilience are considered aspects that cannot be understood only from the perspective of objective data, but rather one has to immerse in its subjective sphere, and the confluence of these two spheres confer reliability to the process this study is intended to analyze. Therefore, the following guiding question is proposed: How do rural women’s perceptions corroborate the results showing that their resilience intersect with their QoL?

In order to answer this question, this study’s objective was to analyze the intersections between rural women’s quality of life and resilience.

## Method

### Study design

This convergent mixed methods design concomitantly implemented qualitative (QUAL) and quantitative (QUAN) elements. These studies were equally prioritized (QUAN + QUAL) but kept independent during the analysis process, while the results were then combined for general interpretation. An implicit perspective of theorization was adopted, anchored on pragmatism, a paradigm proposed for the mixed methods design^8^. 

A cross-sectional QUAN study and a QUAL study guided by oral history were triangulated. Note that data collection and analysis occurred simultaneously but independently, and data were integrated after each study was analyzed separately.

### Study setting

This study was conducted in Nazarezinho, a town located in the backwoods of the state of Paraíba, Brazil. The Brazilian Institute of Geography and Statistics (IBGE) classifies it as a predominantly rural town with approximately 7,280 inhabitants. According to the last demographic census (2010), 4,096 (56.26%) inhabitants lived in rural areas.

### Population

This study’s population comprised 112 rural women enrolled in the micro-area covered by a rural Family Health Strategy facility. This micro-area was chosen by convenience, that is, due to the main author’s familiarity and access to the study setting.

### Selection criteria

The sample comprised women aged 18+ years old who consented to participate and were performing or had performed agricultural labor and/or extractivism at some point in their lives. Women who lived in the rural area but did not present a lifestyle or pursuits related to life in rural areas or lived in the rural area for less than six months were excluded.

### Sampling

A convenience sample without replacement was adopted in the QUAN study. It was calculated with a sample error of 5% and a 95% confidence level, considering a formula^9^ in which N represents the population (112), n_0_ represents an initial approximation of the sample size (400), and E_0_ represents the tolerable sampling error (0.0025), reaching a sample of 87 rural women. 

The participants were selected for the QUAL study based on the concept of oral history of the target community (rural women from Nazarezinho), colony (rural women monitored by a rural FHS facility, living in the study’s micro-area), network, and point zero^10^. The network was determined based on point zero (first interview). The Community Health Agent (CHA) working in the micro-area nominated the first rural woman to be interviewed.

 The rural woman nominated by the CHA (point zero) nominated a second rural woman to participate in the study, who in turn, nominated the following participant until the recruitment of participants ceased, i.e., when the reports enabled answering the study’s questions and became repetitive, as recommended by the framework adopted (Oral History)^11^. The network of collaborators who participated in the QUAL stage was composed of seven rural women.

### Instruments used to collect information

The following instruments were adopted in the QUAN study: socio-demographic form to characterize the sample addressing objective questions related to socio-economic, occupational, and health-disease information. In addition, the Resilience Scale and Medical Outcomes Study 36-Item Short-Form Health Survey (SF-36) were used.

The Resilience Scale measures the level of positive psychosocial adaptation in relevant life events. It was validated for the Brazilian Portuguese and is directed to adult populations. This instrument comprises 25 items rated on a seven-point Likert scale. The higher the score, the more resilient an individual is. For example, scores below 121 points indicate “poor resilience,” while scores between 121 and 145 indicate “moderate resilience,” and scores above 145 points indicate “high moderate” to “high resilience”^12^. 

The SF-36 is designed to assess QoL and was validated in Brazil. The domains assessing QoL include physical functioning (PF), role-physical (RP), bodily pain, general health (GH), vitality, social functioning (SF), role-emotional (RE), and mental health (MH). Its final score (Raw Score) ranges from 0 to 100 points and is calculated for each domain: 0 indicates the worst general health, and a 100 score indicates improved general health^13^.

The QUAL study included open-ended interviews recorded with an mp3 device. The first cue was: “tell me your history in the rural area.” Other cues were presented as the participants shared their histories, such as “tell me more about it” to guide the process.

### Data collection

The main author (a Master’s student) with experience in collecting data and a CHA working in the micro-area (trained by the main author) collected quantitative data between July and November 2020. The interviews were held at the rural women’s homes using a printed form. The main author conducted the qualitative stage by personally interviewing the participants. The interviews had been previously scheduled (pre-interview) via telephone between August and November 2020 and lasted 40 minutes on average.

### Treatment and analysis of data

Quantitative data were typed in electronic spreadsheets and submitted to descriptive and inferential statistical analysis using IBM SPSS Statistics® version 25.0. The Kolmogorov-Smirnov test was initially used to analyze whether data were normally distributed. Associations between the SF-36 domains and level of resilience were associated using the Mann-Whitney’s U test. Hence, a nominal variable was created based on the mean score of resilience, moderate level of resilience (121 to 145 points obtained in the Resilience Scale). IT was used as a grouping variable in the software, resulting in two sample groups: one group with a moderate level of resilience and one group not presenting a moderate level of resilience.

The relation of dependence between QoL domains and level of resilience was analyzed with a multiple regression linear model, in which the level of resilience was the dependent variable, while the eight domains of QoL were independent variables. Note that all the analyses were conducted with a level of significance established at 5% (p-value<0.05).

Data produced in the qualitative analysis were transcribed verbatim, followed by textualization and transcreation, a stage in which the texts of the interviews were returned to the participants for them to review and legitimate. After the participants’ feedback (the participants did not change the texts), the histories of life were treated using inductive thematic analysis, a method used to identify, analyze and report patterns (themes) within a set of data, enabling its organization and description with rich detailing. In this approach, coding (manually performed by the main author) does not assume a previously established theoretical framework; i.e., analysis was guided by data^14^. 

The results were integrated by developing a convergences diagram and a joint-display table. Hence, meta-inferences of mixed methods were obtained, which resulted from combining quantitative and qualitative inferences. Finally, the results were interpreted from the perspective of gender, resilience, and QoL.

### Ethical aspects

All ethical guidelines provided by resolutions 466/2012 and 510/2016, Brazilian Council of Health, were complied. Furthermore, the study’s objectives were presented to the participants before they signed (two copies) free and informed consent forms. The study was approved by the Institutional Review Board at the Federal University of Rio Grande do Norte (UFRN), opinion report 3,950,023. Pseudonyms were used to ensure the identity of the qualitative study’s participants remained confidential. Hence, common rural names were adopted, followed by the name “Margarida” [Daisy in English] in honor of Margarida Maria Alves, a rural woman from Paraíba who dedicated her life to enabling country people to have access to land.

## Results

A sample of 87 rural women participated in the QUAN study. Most of these women reported being Caucasian (48.3%), married (60.9%), with incomplete primary education (64.4%), and aged 50.15 years old on average. The following age groups were identified: 12.6% (n=11) were between 18 and 30 years old; 13.8% (n=12) were 31 to 40; 19.5% (n=17) were between 41 and 50; 27.6% (n=24) between 51 and 60; and 26.4% (n=23) were 60+ years old.

Regarding labor-related aspects, most (70.1%) participants were farmers for at least ten years, with planting being the most recurrent activity (95.4%). However, note that even though most of the participants farmed for a long time, practically none (97.7%) received any pay (except for pension).

Regarding the social and clinical aspects and health-disease continuum of the women participating in the QUAN study, almost half (47.1%) reported a chronic disease; systemic hypertension was the most common (29.9%). None of the participants had private health insurance/plan and health services were mostly (93.1%) provided by Primary Health Care (PHC Units). Regarding social aspects, most (98.9%) rural women reported they felt welcomed by their communities.

The women participating in the QUAL study was 67 years old on average, most were married (71%), had incomplete primary school (57%), reported being Caucasian (57%), and a monthly income of two times the minimum salary (current minimum wage = R$1,045.00, Brazil, 2020).

Regarding the quantitative results, a moderate level of resilience ( =129.14) was found, minimum 103 and maximum of 164 points; the highest scores were obtained in the PF and SF domains. Table 1 presents the maximum and minimum scores and the mean scores of the QoL domains assessed by SF-36, the result of the test measuring associations between them, and the level of resilience was found (moderate). In addition, a statistically significant association was found between resilience and the SF domain.

**Table 1:** Minimum, maximum and mean scores of quality of life and association with a moderate level of resilience among rural women. Nazarezinho, PB, Brazil, 2020

Variable	Minimum	Maximum	Mean	p*
PF	30	100	77.36	0.445
RP	0	100	55.46	0.675
Bodily pain	0	100	62.64	0.304
GH	10	95	53.74	0.631
Vitality	25	95	68.28	0.426
SF	25	100	73.22	0.046
RE	0	100	61.68	0.184
MH	28	100	72.97	0.236

*Mann-Whitney

Regarding the regression model in which the level of resilience was a dependent variable, and the SF-36 eight domains were independent variables, it predicted 14.1% of the variance in the level of resilience (R2=0.141; F=1.60; p=0.004). The statistically significant association between the level of resilience and the QoL SF domain, together with the result obtained in the regression analysis, indicates that the constructs QoL and resilience mutually influence each other.

As for the qualitative results, the inductive thematic analysis of the histories of life enabled extracting the main themes that emerged in the analysis: rural life as a promoter of resilience and QoL; inequalities and iniquities experienced in rural life; extenuating rural labor; conceptions of health and QoLrelated to the rural social imaginary; and protective factors promoting resilience and QoL, such as spirituality, social support networks, and feelings of belonging and enchantment with the rural life. The following excerpts portray these themes:

*I experienced many hardships, to the point of not having clothes or shoes to wear, or not even having a hammock to sleep* (Severina Margarida)*; [...] I planted corn, beans, rice, and we harvested the corn ourselves. I didn’t know how to drill [prepare the soil], but would dig a hole for a fence, stretch wire, handled the grazing cattle, we’d do all that* (Raimunda Margarida).;

*[...] I have arthritis, osteoarthritis, spine problems, and I guess these problems were caused by lifting too much weight* (Tereza Margarida).;

*For me, being healthy, I don’t think it means not feeling anything, I guess people sometimes believe they are healthy because they feel no pain, and sometimes, it’s not like that, sometimes a person may have severe problems and doesn’t know. I believe that living healthy is it, thinking that you are well [...]* (Severina Margarida).;

*[...] I’ve always liked the countryside… because it’s good! You see… you plant, and you reap… you reap the fruit of your work, to this day, I think it’s very satisfying [...] you watch plants sprouting... it’s very gratifying! I think it’s a gift! You have to enjoy it… and I have this gift, I enjoy it! [...] this interaction with nature, it changes you, makes you stronger to overcome stuff [...]* (Tereza Margarida).;

*[...] on the farm you feel calm, you trust the people you know, we help each other in times of need [...]* (Francisca Margarida).

The joint display represented by Figure 1 presents the integrated results and highlights some excerpts of the inductive thematic analysis portraying the themes unveiled by the qualitative analysis and a summary of the quantitative associations. Next, meta-inferences are proposed based on the inferences obtained in each isolated approach. These findings enabled a better understanding of how the two constructs, resilience, and QoL, influence each other, indicating various protective factors arising from these women’s rural context that contribute to the development of resilient processes and positively influence their QoL, consequently reflecting and feeding back their health and resilience.

**Figure 1:** Joint display of the quantitative and qualitative inferences and mixed method meta-inferences concerning the intersections between the rural women’s level of resilience and quality of life. Nazarezinho, PB, Brazil 2020

Main topic	QUAN results	QUAL results	Combination of mixed method
**Integration**	**Associations between the level of resilience and the SF-36 domains (Mann-Whitney test)**	**Themes and excerpt from the thematic analysis**	**Meta-inferences**
Intersections between resilience and QoL	No statistically significant association was found between the level of resilience ( =129.14) and the SF-36 (p=0.445) PF domain* ( =77.36)	*Rural life as a promoter of resilience and QoL [...] we got here, as the history says, at our age today, we’ve suffered a lot in the country, worked a lot, as men did, you know? But everyone was happy, I was happy, I won’t say I wasn’t, I think I was happier than I am today [...]* (Joaquina Margarida).	*Divergence* The women reported that physical strain demanded by rural labor is a positive intervenient factor in their health and QoL (even though work has also been related to disease), and being enchanted with the rural life was a protective factor for the development of resilience, which conflicts with a lack of statistical significance between the level of resilience and the SF-36 PF domain found in the Mann-Whitney test.
	No statistically significant association was found between a moderate level of resilience ( =129.14) and the SF-36 (p=0.675) RP domain^†^ ( =55.46)	*Rural life as a promoter of resilience and QoL [...] You may note that people like me, who’ve worked in the countryside, are strong, won’t fall easily [...]* (Francisca Margarida).	*Divergence* The rural women’s concepts indicate that even though they faced limitations that impeded their activities, they were able to prevail, revealing resilience even when dealing with obstacles, which also conflicts with the lack of statistical significance found in the Mann-Whitney test.
	No statistically significant association was found between a moderate level of resilience ( =129.14) and the SF-36 (p=0.304) pain domain ( =62.64)	*Rural life as a promoter of resilience and QoL [...] even now I feel like going to work in the countryside... but I can’t because of my pains [...]* (Sebastiana Margarida).	*Divergence* Bodily pain stands out as a factor that limits everyday activities of these rural women, impeding them from doing their work, though they see it as a source of resilience so that their pain is a fact that negatively interferes in their QoL. This finding also conflicts with the lack of statistical significance in the Mann-Whitney test.
	No statistically significant association was found between a moderate level of resilience ( =129.14) and SF-36 (p=0.631) GH domain‡ ( =53.74)	*Concepts on health and QoL My health is terrible, but I make it seem not so terrible because when I have problems, I put them in God’s hands and ask Him to give me strength to overcome them [...]* (Tereza Margarida).	*Divergence* Protective factors such as spirituality promote resilience among rural women enable them to develop coping strategies to deal with unfavorable health conditions, also differently from what was found in the Mann-Whitney test, which did not present any statistical significance between the level of resilience and the SF-36 GH domain.
	No statistically significant association was found between the level of resilience ( =129.14) and the SF-36 (p=0.426) vitality domain ( =68.28)	*Concepts on health and QoL When I used to work in the countryside, I had quality of life, health and courage; I worked in the field on Saturday many times, sometimes there were dance parties on Saturday night, and I’d go, and was brave enough. I guess that people who work in the countryside are healthier (*Sebastiana Margarida).	*Divergence* Bravery, a synonym of vitality and energy, is alluded by the rural women as coming from their everyday work, permeated by protective factors that culminate in resilient processes, also contrasting with the lack of statistical significance verified by the Mann-Whitney test.
	No statistically significant association was found between a moderate level of resilience ( =129.14) and the SF-36 (p=0.046) SF domain^§^ ( =73.22)	*Rural life as a promoter of resilience and QoL [...] There is tranquility on the farm, you trust the people you know, we help each other in times of need [...]* (Francisca Margarida).	*Convergence* The establishment of social support networks with the community was a protective factor revealed in the reports of rural women, which promoted resilience and QoL, corroborated by the statistical significance found in the Mann-Whitney test.
	No statistically significant association was found between the level of resilience ( =129.14) and the SF-36 (p=0.184) RE domain^||^ ( =61.68)	*Rural life as a promoter of resilience and QoL [...] Whenever I have problems, I put them in God’s hand and ask Him strength to overcome them [...] so, I’ve always found a solution for my problems* (Tereza Margarida).	*Divergence* Protective factors such as spirituality and hope (closely linked to the role emotional domain) promote resilience that leads rural women to overcome daily challenges and enjoy greater QoL, which diverges from the lack of statistical significance found in the Mann-Whitney test.
	No statistically significant association was found between a moderate level of resilience (=129.14) and the SF-36 (p=0.236) MH domain^¶^ (=72.97)	*Rural life as a promoter of resilience and QoL I’m anxious, but when something makes me anxious, I deal with it, you know? I’m strong, won’t fall apart… Even if I’m sick, I won’t break easily, you have to hold your head up high, and whenever you have a problem, always see both sides [...]* (Raimunda Margarida*).*	*Divergence* Rural women find ways to live with quality based on their hope and resilient attitude, despite their psychosocial problems like anxiety, and keep their level of psychological well-being by facing obstacles from more than one perspective. However, the association between resilience and mental health was not statistically significant in the Mann-Whitney test.
	**Relationship between the level of resilience and the SF-36 domains (multiple linear regression)**	**Themes and excerpts from the thematic analysis**	**Meta-inferences**
	The linear regression analysis with the SF-36 domains as independent variables predicted 14.1% of the variance in the level of resilience, which was a dependent variable (R^2^=0.141; F=1.60; p=0.004).	*Rural life as a promoter of resilience and QoL I guess I’m resilient because I’ve suffered a lot where I came from [...] I experienced many hardships and obstacles, but I thank God for going through all these; I guess that I’m an overcomer. I’ve faced so much, but still, I moved on* (Tereza Margarida).	*Magnification* The rural women’s understanding shows that the adversities they face in their daily lives and work contributed to their health and QoL, working as protective factors that promote resilience processes. The QoL domains in the regression model corroborate these resilience processes, which positively feedback these women’s health.

*Physical functioning; ^†^Role-physical; ^‡^General health; ^§^Social functioning; ^||^Role-emotional; ^¶^Mental health.

**Figure 2:**
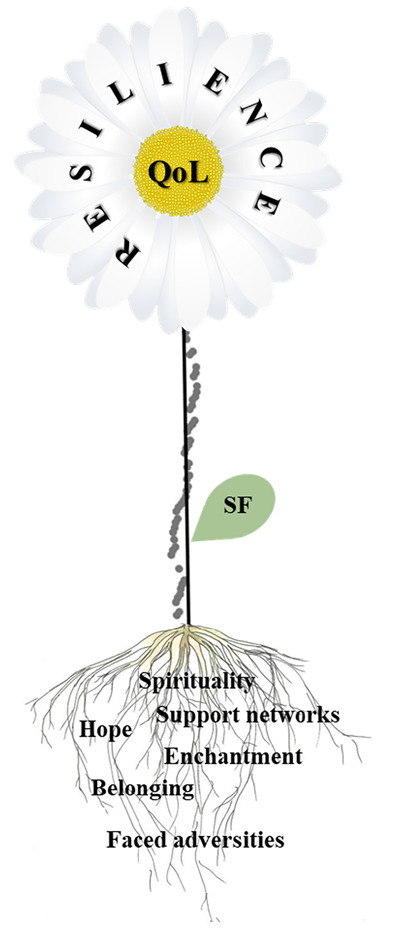
Convergences that represent the influence of resilience on the rural women’s quality of life. Nazarezinho, PB, Brazil, 2020

In order to organize convergences that resulted from the influence of resilience on the QoL of rural women, we developed a diagram of convergences, represented by Figure 2. The figure was represented in the form of a daisy, in which the main protective factors expressed in the themes were extracted from these women’s reports and resulted in the formation of resilient processes, were represented as the daisy’s roots, as they are the basis of the process.

The diagram’s stem represents an adaptation of the residue graph of the multiple linear regression, while a single leaf represents the social functioning domain of QoL because it was the only domain that appeared significantly associated with resilience. The roots and stem represent the structure that sustains the daisy, and its flowering represents the positive influence that resilience exerts over the QoL of rural women.

## Discussion

The mean score (moderate) obtained for resilience could not be compared with the literature because there are no Brazilian or international studies assessing the relationship between resilience and QoL among rural women. However, a study conducted in Portugal^15^, using another resilience scale, reports a moderate level of resilience among rural elderly individuals, though the analysis did not consider gender differences. Other researchers^16^, using the same instrument, identified a moderate level of resilience among individuals living in rural areas affected by drought.

The moderate level of resilience identified in this study was similar to that found in a sample of the Brazilian population composed of 2,038 individuals, in which researchers^17^ found a moderate level of resilience of approximately 124.60 points. In addition, the analysis did not consider differences between genders and indicated that higher levels of resilience were associated with higher educational levels. However, this study’s moderate level of resilience might be related to a lower level of education and older age.

Note that even though the mean score indicates a moderate level of resilience, there is a need to invest in strategies and interventions to enable rural women to follow paths other than farm work, strengthening and promoting their resilience. These interventions are not supposed to romanticize inequalities and inequities but emphasize that resilience helps them promote courage, skills, and confidence to main their health even when their context favors marginalization and vulnerability^18^.

Researchers^19^ indicate that the individual level of resilience varies depending on the individuals’ characteristics and their social context, directly contributing to QoL. Consequently, resilient individuals experience improved QoL arising from their ability to deal with and overcome hardships and general problems more easily^6^.

Regarding QoL, the worst mean scores were obtained in the GH and RP domains, similar to the scores reported by a cross-sectional study conducted with 355 rural workers in the state of Bahia, Brazil, which indicated demographic and socioeconomic variables as related motives^20^. In addition, studies note that rural communities present the lowest levels of QoL^4^ ^,^ ^21^, revealing that these communities are often and historically overlooked by public policies and health care models, directly affecting this population’s QoL^22^.

Characteristics such as age, poor education, and lower socioeconomic levels are associated with lower levels of QoL, showing that the poorest and less educated individuals are the most vulnerable groups. Additionally, the older an individual, the more negative his/her QoL is because of the relationship between age and the emergence of various morbidities and limitations^4^.

As for the conceptions of health and QoL expressed by the rural women in the QUAL study, health in the rural context can be understood as an ability to perform everyday tasks, while health needs are secondary to work needs. This study also shows that health is represented as the ability to overcome adversities and is related to the resilience concept itself^23^.

From this perspective, having knowledge of resilience in the occupational health field, in this case, rural workers, can support a better understanding of these workers’ environment, helping them maintain or recover their health, which results in QoL^24^. The strategies that can be implemented in interventions to improve the QoL of rural women include improving family income and facilitating access to high-quality health care services^25^. 

Likewise, the implementation of public health programs intended to improve the QoL of the rural population is essential, considering that it is an indicator that produces information capable of identifying and screening this population’s health needs^4^. Thus, public agencies should implement specific programs directed to rural workers to promote their integral health^26^.

Therefore, even though quantitative findings indicate that resilience only influences one of the QoL domains, the qualitative results unveil divergences, enlarging an understanding that this influence may not be quantifiable. Additionally, the regression analysis showed the influence of the eight domains of QoL (SF-36) on the level of resilience, indicating the mutual influence of these constructs, as reported by a study developed with adolescents^27^.

Even though there are no other studies specifically reporting the influence of resilience on rural women’s QoL, the association of these two constructs was found in other studies among different populations. For example, a study with 108 elderly individuals with chronic pain was conducted in São Paulo, Brazil, and verified that those with the highest levels of resilience also experienced improved QoL. Additionally, higher levels of resilience positively anticipate QoL^28^. Another study^29^ found a synergy between resilience and QoL among adolescents in Cuenca, Ecuador.

The meta-inferences proposed by this study and reinforced by studies developed in different contexts addressing different populations suggest that the QoL of rural women is influenced by their level of resilience, which is favored by protective factors. Hence, these factors culminate in resilience processes that promote QoL and are seen as processes that support changes and positive adaptation when facing risks. These processes may be individual (positive self-esteem), at the family level (such as stable relationships), or social support (support networks)^30^.

Social support networks stood out among these factors and were confirmed by the reports of the women participating in the QUAL study. Most of the women in the QUANT study also reported a sense of being welcome, and an association was found between a moderate level of resilience and the SF domain of QoL. Social support is essential for developing resilience and improving QoL^31^.

Despite the role these networks play, the low score obtained in the SF-36 RE domain may be explained by the fact that this domain assesses the repercussions of the psychological characteristics on the individuals’ wellbeing, which are individualized and subject to the influence of other domains that also obtained low scores, such as GH, RP, and bodily pain, which, unlike the SF domain, assess the integration of the individuals in social activities^13^. 

Religious beliefs and spirituality were also identified as protective factors linked to the fact that these individuals’ lives are permeated by a transcendental dimension (which is not necessarily connected to a religion or spirituality), producing in people a feeling of belonging to something higher, timeless, and unlimited that produces an existential meaning^32^.

Spirituality is a factor that can be used in times of crisis to facilitate the process when transitioning to a healthy adjustment, considering that believing in something higher has a calming effect, attenuating a negative emotional load arising from adverse events, enabling individuals to more effectively manage events that could break one’s Self^33^.

Other protective factors the women reported included having hope in the work they perform, a finding that other researchers^34^ highlight, and being enchanted with farming, summarized as a project of work and life, valuing the farmers’ customs and knowledge in the development process, encouraging their dream of living with freedom and the desire to cultivate their own land, master their work, and live with their families in the countryside with dignity^35^.

However, despite this enchantment, even though the conception of resilience adopted here is not intended to justify the inequalities rural women experience, who engender the resources needed to face their work (which despoils them) with resilience, this enchantment is admittedly idealized. Hence, there is a need to discuss these aspects from the perspective of gender, which emerges in social sciences to define identities, values, roles, representations, and/or symbolic feminine and masculine attributes not as an effect of “nature”, but as the product of different ways of socialization produced and reproduced by the individuals, highlighting the asymmetry of social relations between men and women^36^.

From this perspective, it is apparent that starting in adolescence, men are more likely to achieve higher levels of education and are expected to inherit their families’ rural possessions, while women remain as caregivers^37^. 

Even though women’s participation in the world of work is gradually increasing, this division still exists and reaffirms the sexual division of labor that overlook the work of women, delegating to them specific cultural roles: that of caring serving, which express socio-cultural representations of the society’s imaginary and often leads rural women to omit their individual histories and everyday lives, disregarding their aspirations, so that they adapt or reproduce roles constructed by social representations of the roles of homemaker and maternity^38^.

This invisibility is incorporated in the female rural imaginary and leads women to devalue their own work, even though they perform the same tasks men perform, such as their fathers and brothers, in addition to the housework. Even if wives and daughters farm, they are considered mere helpers or are seen as the “producer’s wife,” and when they are paid, they are not allowed to decide about the money they receive^39^ ^-^ ^40^, which is confirmed by this study; approximately 97.7% of the rural women who participated in the QUAN study did not receive pay for their rural labor.

However, even though women are linked to the reproductive domain (housework and childcare), in a rural context, women are also active in the productive domain, i.e., cultivation and farming, which is often attributed to men. Thus, women have responsibilities in the two spheres, which leads to work overload^39^. 

Even though the challenges and adversities of the farming life are acknowledged, this is a fact that rural women considered to be a source of strength and joy for being able to overcome adversities, which appears as a resilient behavior to overcome challenges, which when overcome, promote even greater resilience, as the reports show.

However, even though the fact mentioned above works as a protective factor, gender roles are implicated in their enchantment with farming because rural women generally do not have a choice about what work they will perform, i.e., they are introduced to the context of agricultural activity since their childhood^34^.

Researchers^41^ suggest that enchantment may be related to the symbolic value assigned to labor, originated from the social imaginary of being inserted in the productive chain, and feeling beneficial to society, which confers status. It may be reinforced by the association the rural women made between farming and freedom and autonomy, being satisfied for not being submitted to urban standards and pursuits, valuing and being guided by nature, and perceiving that they are transforming their context^34^. 

Even though farming renders women vulnerable, they find meaning in their work, which influences their QoL^42^. Nevertheless, rural women need to be empowered to face their work beyond enchantment and broaden their knowledge regarding their contexts to propose alternatives and consider it an important instrument to improve their life conditions^43^.

The conception of belonging to one’s social context was also evidenced as a protective factor, which, in addition to being linked to support networks, portrays a feeling that is surrounded by close bonds established with their community and which is strongly related to the identity of rural women, also reported by other studies^44^.

From this perspective, individuals living in cities, for instance, do not understand the feeling of belonging that rural people experience because they do not have their roots in the rural area and do not put their hands and feet on the land, which impedes the establishment of a relationship with the territory they inhabit^45^. 

The protective factors identified in the reports of rural women can be used in psychosocial interventions to promote resilience and QoL and support public policies intended to promote QoL. Rural nurses play an essential role in this process because they are very familiar with the histories of life of the individuals under their care and, consequently, are aware of the protective factors they develop when facing adversities^46^.

This study’s limitations include the application of instruments to collect quantitative data. Even though validated instruments were used, the rural women had difficulties understanding the instruments, showing a need to perform a cultural adaptation of these instruments to the rural population. Additionally, the small sample addressed in the QUAN study hindered the generalization of results.

## Conclusion

This study analyzes the intersections between QoL and resilience among rural women, showing that when these women develop protective factors, they also become more resilient, a factor that influences and is influenced by their QoL. These factors include spirituality, establishing social support networks, and a feeling of belonging. 

The results reinforce the need for a more effective work of FHS, a healthcare modality that directly works in the territory of rural women, in promoting health, the purpose of which is to enable rural workers to improve their QoL. In addition, the care provided by the FHS should strengthen and include attentive listening when providing care to rural women, an instrument that enables identifying protective factors and supporting psychosocial interventions to promote more resilient attitudes, which can improve QoL.

This study’s results contribute to the practice of rural nurses, who can organize care delivery by incorporating protective factors developed by rural women and promoting these factors in different opportunities: prenatal nursing consultations, consultations addressing growth and development, during home visits, and group actions, which can contribute to qualify and make them more congruent to the needs of the rural clientele.

Note that the influence of the constructs found in this study would not be understood if only one approach was employed - quantitative or qualitative - highlighting the contribution of this mixed methods design to understanding complex and imbricated phenomena, such as QoL and resilience. The only quantitative association found and limited prediction level could have led to erroneous interpretations regarding the intersections between the constructs.

Note that cultural aspects and the participants’ level of education limited the participants’ ability to answer the instruments in the quantitative stage, a limitation overcome by individualized interviews based on Oral History. Hence, the mixed methods design validated the results presented in this investigation.
